# Maternal and Neonatal Outcomes in Women with Metabolic Syndrome and Substance Use Disorder

**DOI:** 10.3390/life13091933

**Published:** 2023-09-19

**Authors:** Vijaya Lakshmi Sundaram, Rajan Lamichhane, Alfred Cecchetti, Subha Arthur, Usha Murughiyan

**Affiliations:** 1 Department of Clinical and Translational Sciences, Marshall University School of Medicine, 1600 Medical Center Drive, Huntington, WV 25701, USA; 2 Department of Internal Medicine, Marshall University School of Medicine, 1600 Medical Center Drive, Huntington, WV 25701, USA

**Keywords:** metabolic syndrome (MetS), substance abuse disorder (SUD), pregnancy complications, neonatal outcomes, logistic regression model

## Abstract

Introduction: Metabolic syndrome amplifies the risk of gestational diabetes, preeclampsia, and preterm labor in pregnant women. Similarly, women with substance use disorder have worsened obstetric and birth outcomes. Despite these two conditions being major healthcare disparities in Appalachia, the health outcomes of this cohort have not been studied thus far. This study looks at the health outcomes of this cohort. Method and Results: In this retrospective cohort study, we analyzed 27,955 mothers who delivered at Cabell Huntington Hospital between January 2010 and November 2021. We implemented Chi-square tests to determine the associations and multiple logistic regression methods for comparison after controlling for other factors, and found that MetS, together with SUD, significantly increases the risk as well as the number of pregnancy complications such as gestational diabetes (*p*-value < 0.001), preeclampsia (*p*-value < 0.001), premature rupture (*p*-value < 0.001), preterm labor (*p*-value < 0.001), and newborn disorder (*p*-value < 0.001) compared to the women who had none or had either MetS or SUD alone. Conclusion: Women with both metabolic syndrome and substance abuse had worsened pregnancy and neonatal outcomes compared to women with metabolic syndrome or SUD alone. In conclusion, analysis of all the variables is crucial to strategically planning and implementing health interventions that will positively influence the health outcome of the pregnant woman as well as the child.

## 1. Introduction

Appalachia in general is plagued with numerous healthcare disparities. West Virginia is the only state that is entirely situated within Appalachian borders. In addition to health care disparities, West Virginia is the epicenter of the drug epidemic, with the highest rates of overdose across the nation [1]. There were 1211 overdose deaths involving opioids between January 2020 and January 2021; a 10% increase in opioid overdose deaths was reported between December 2020 and December 2021 [2]. This high incidence of healthcare disparities and drug addiction can be attributed to a combination of sociocultural factors, low socioeconomic status, lack of education, and high rates of prescription and dispensing of substance abuse drugs. Providers in West Virginia wrote 69.3 opioid prescriptions for every 100 persons, compared to the average U.S. rate of 51.4 prescriptions. In 2017, USD 7247 per capita was spent on the combined costs of opioid use disorder and fatal opioid overdoses—131% more than the national rate [3,4]. Specifically, low-income individuals (on Medicaid and others) were regarded to be at high risk for prescription drug overdose as per the Centers for Disease Control and Prevention (CDC) report [5]. The prevalence of obesity in West Virginia is 40.3%, which is the highest in the nation, with more than two-thirds (70.9%) of adults being considered overweight or obese [6]. Additionally, two out of three women (66.9%) in the United States are overweight or obese [7].

Metabolic syndrome (MetS) is a term proposed by the World Health Organization (WHO) in 1998 as a replacement for insulin resistance syndrome to encompass the combined risk factors associated with type 2 diabetes mellitus [8]. MetS, consisting of complex abnormalities including abnormal abdominal obesity, dyslipidemia, hypertension, and diabetes, is caused by obesity, which is one of the major contributors to healthcare disparities in Appalachia. MetS increases the risk of heart disease, stroke, and cancer. It has been shown that Appalachian women have a higher mortality rate due to cardiovascular disease and stroke [9]. Studies have also shown that MetS amplifies the risk of developing gestational diabetes, preeclampsia, and preterm labor in pregnant women [10]. Every year, 2 to 10% of pregnancies are affected by gestational diabetes [11]. Researchers in one study found that the residents of 78 counties classified as distressed (lowest on a five-point socioeconomic scale) were 1.4 times more likely to have diabetes than residents of non-Appalachian counties [12]. It has also been shown that women with gestational diabetes carry a lifetime risk of progression to type 2 diabetes of up to 60% [13] and a twofold higher risk of future cardiovascular conditions [14]. Women with preeclampsia have an increased risk of ischemic heart disease. By identifying MetS in pregnant women who are at a higher risk of developing these pregnancy-related complications, namely, gestational diabetes, preeclampsia, hypertension, and preterm labor, we can implement management strategies to improve the health outcomes of pregnant women. There have not been many studies conducted on pregnancy complications among Appalachian women with metabolic syndrome.

According to the national comorbidity survey replication study, substance use disorder affects a significant minority of the general population [15,16]. Over the past decade, substance use has steadily increased [17]. In rural Appalachia, abuse of prescription medications like opioids has exceeded the national average [18]. The highest rates of use and overdoses across the US are found in counties within West Virginia, Southwest Virginia, Eastern Kentucky, Southeast Ohio, East Tennessee, and Western North Carolina [19]. Currently, WV has the highest rate of drug overdoses in the country, with 35.5 overdoses per 100,000 inhabitants compared to a national average of 14.7 per 100,000 people [20]. Substance use during pregnancy is of concern for both maternal and fetal health. Cocaine and heroin are not the only things that can cause substance abuse. Legally obtainable substances like tobacco, benzodiazepines, amphetamines, and alcohol have the potential to cause addiction. It is reported that among pregnant women, 18% have smoked tobacco, 9.8% have consumed alcohol, and 4% have used illicit drugs during pregnancy [21]. Studies have also shown that 93% of pregnant women using cocaine or opioids also use other drugs that are known to cause health risks to the fetus [20]. Women who abuse substances during pregnancy have a higher maternal risk of developing medical complications like syphilis, gonorrhea, hepatitis, septicemia, and pregnancy-related complications like preterm labor, abruptio placenta, miscarriage, bleeding in the 3rd trimester, seizure, preeclampsia, and stillbirth [22,23,24,25]. They are also at a higher risk of the fetus developing low birth weight, heart problems, low Apgar score, stroke, sudden infant death syndrome (SIDS), neonatal abstinence syndrome (NAS), fetal alcohol syndrome, etc. WV, which serves the tri-state area of WV, KY, and OH, has demonstrated that pregnant women suffering from drug addiction also tend to be obese. About 30% of overweight or obese women in this country consume alcohol regularly which contributes to a higher risk of these pregnant women developing MetS [26]. There are several health risks such as cancer, fatty liver disease, hypertension, heart disease, hepatitis B, hepatitis C, and HIV that are associated with obesity and substance abuse. Researchers are beginning to understand the relationship between substance abuse and obesity. It is not yet clear whether obesity leads to substance abuse or vice versa; nonetheless, there is clearly a complex relationship between the two conditions. There have been a few studies conducted on pregnant women who are obese with MetS and pregnant women who have a history of substance abuse separately. However, despite these two conditions being major healthcare disparities in Appalachia, the health outcomes of pregnant women with MetS and drug addiction in conjunction have not been studied thus far. Therefore, in this study, we seek to determine the complications during pregnancy among women suffering from MetS and substance abuse disorders. By identifying the possible modifiable conditions among this cohort of women, we hope to improve the health outcomes of this cohort during pregnancy. This study will add new dimensions to maternal morbidity research by incorporating a large pool of data from the unique population of underprivileged rural Appalachia. It will open a new area of research that could reduce the risk of pregnancy complications among women who suffer from both MetS and substance abuse.

## 2. Materials and Methods

This retrospective cohort study included data from the Translational Science Core Data Warehouse on 27,955 mothers whose corresponding deliveries took place at Cabell Huntington Hospital between January 2010 and November 2021. The study population was primarily located in Central and North Central Appalachia, which includes the western part of West Virginia, the southern part of Ohio, and the eastern part of Kentucky. Pregnant mothers were classified as with or without metabolic syndrome if they met at least 3 of the following 5 conditions immediately before or at the time of the birth of the baby: (1) insulin resistance (IR): average fasting glucose ≥ 100 mg/dL or HbA1c ≥ 5.7 at any point, or receiving drug therapy for hyperglycemia or type 2 diabetes listed as a billing diagnosis or under a problem list; (2) hypertension (HTN): blood pressure ≥ 130/85 mm Hg or receiving drug therapy for hypertension or hypertension listed as a billing diagnosis or under a problem list; (3) hypertriglyceridemia (HG): average triglycerides ≥ 150 mg/dL or receiving drug therapy for hypertriglyceridemia or hypertriglyceridemia listed as a billing diagnosis or under a problem list; (4) low high-density lipoprotein cholesterol (HDL): average HDL-C < 40 mg/dL in men or < 50 mg/dL in women, or receiving drug therapy for reduced HDL-C or low HDL-C listed as a billing diagnosis or under a problem list; and (5) obesity: average BMI > 30, or obesity listed as a billing diagnosis or under a problem list.

Another important characteristic we considered was substance use disorder (SUD). This was determined based on whether the patient had a diagnosis of SUD or had a positive urine drug screen any time before the delivery date. We further categorized the data based on the following MetS and SUD statuses. MetS alone: Patients who had at least 3 of the above-mentioned MetS components and who did not have SUD; SUD alone: Patients who had SUD but not MetS; SUD + MetS: Patients who met both the SUD and MetS criteria; No MetS or SUD: Patients who presented no SUD and did not meet the MetS conditions.

The following five complication outcome variables were documented as diagnoses or in problem lists during pregnancy (within 42 weeks, before the birth of the baby): gestational diabetes, preeclampsia, premature rupture, pre-term labor, and newborn disorder (whether the newborn had a diagnosis of a disorder related to short gestation or fetal growth). There were not enough data to study maternal death (death within 7 days following the birth of the baby). We further grouped the outcome into the number of complications based on the previously mentioned 5 complications: one complication, two complications, three complications, four complications, or five complications.

The other study variables included in the study were the following: (1) demographic variables—maternal age at the time of birth (in years); (2) body mass index (BMI)—Current BMI was used due to the unavailability of waist circumference in the data; (3) smoking history, which was documented as ex-smoker or smoker; and (4) alcohol use.

To further evaluate the complications associated with MetS and SUD, we categorized individuals into MetS alone, SUD alone, both SUD and MetS, and none of the above. Since multiple complications could exist at the same time, we created complication categories, i.e., at least one, at least two, at least three, and at least four complications, based on gestational diabetes, preeclampsia, premature rupture, pre-term labor, and newborn disorder, and analyzed how MetS and SUD could elevate the risk of these complications. We implemented the Chi-square test of associations to identify the association between complications and MetS and SUD, and then further implemented the logistic regression analyses and calculated odds ratios to compare the odds of complications. The additive effects due to the interaction between SUD and MetS were also estimated using relative excess risk due to interaction (RERI) analysis. Statistical analyses were conducted using SAS version 9.4 (SAS Institute). All *p* values < 0.05 were considered statistically significant. Epidemiologic research using our current data was reviewed and approved by the institutional review board at the Joan C Edwards School of Medicine, Marshall University.

## 3. Results

This study included data from the Translational Science Core Data Warehouse on 27,955 mothers whose corresponding deliveries took place at Cabell Huntington Hospital between January 2010 and November 2021. There were 34.5% obese women in the sample, with an average BMI of 30.25. The average age at birth was 26.40 years, and almost 46% of the women had been prescribed opioids at least once before or at the time of birth during pregnancy. These figures are consistent with the regional trend. Table 1 shows the summary of the data.

There is a significant association between MetS, SUD, and pregnancy complications and newborn disorder (all *p*-values < 0.001) (Table 2). There was a significant increase in gestational diabetes, preeclampsia, pre-term labor, and newborn disorder among women who had three or more MetS components compared to the control (those who did not have both MetS and SUD). Similarly, pregnant women with SUD also had higher complications, including preeclampsia, premature rupture, pre-term labor, and newborn disorder, compared to the control group. Interestingly, we observed decreased premature rupture among the MetS group and decreased gestational diabetes among the SUD group compared to the control. The combination of MetS and SUD had all complications elevated (Table 2).

Metabolic syndrome together with SUD significantly increases the risk of pregnancy complications such as gestational diabetes (*p*-value < 0.001), preeclampsia (*p*-value < 0.001), premature rupture (*p*-value < 0.001), preterm labor (*p*-value < 0.001), and newborn disorder (*p*-value < 0.001). Due to the small sample size, we are unable to assess the risk of maternal mortality (only four mothers died within 7 days following the birth of the baby).

We compared the pregnancy complications between different groups of pregnant women—MetS alone, SUD alone, and both MetS and SUD—by using logistic regression models (Table 3). Women who had both MetS and SUD had significantly higher odds of preeclampsia, preterm labor, and newborn disorder compared to the women who had MetS only or SUD only. Compared to the women who had MetS only, women who had both MetS and SUD had 55% higher odds of developing preeclampsia (*p*-value < 0.001), 141% higher odds of having preterm labor (*p*-value < 0.001), and 96% higher odds of newborn disorder (*p*-value < 0.001). Similarly, compared to the women who had SUD only, women who had both MetS and SUD had 672% higher odds of developing gestational diabetes (*p*-value < 0.001), 245% higher odds of developing preeclampsia (*p*-value < 0.001), 26% higher odds of preterm labor (*p*-value = 0.0278), and 46% higher odds of newborn disorder (*p*-value = 0.0007). We observed that the interaction between SUD and MetS led to an additive risk of preeclampsia, but we did not see this effect with other complications.

We also noticed an increasing trend in the number of complications among pregnant women who had MetS and SUD compared to the women who had MetS alone or SUD alone. Women with both MetS and SUD both were at significantly higher risk of developing multiple pregnancy complications compared to the women with MetS or SUD alone. By our definition, gestational diabetes, preeclampsia, premature rupture, preterm labor, and newborn disorder are considered pregnancy complications. Compared to the women with SUD alone, women with both MetS and SUD had 155% higher odds of developing one pregnancy complication (*p*-value < 0.001), 276% higher odds of developing two pregnancy complications (*p*-value < 0.001), 315% higher odds of developing three pregnancy complications (*p*-value < 0.001), and 1304% higher odds of developing four pregnancy complications (*p*-value < 0.001). The interaction between SUD and MetS had an additive effect. The risk of at least two or more complications was significantly higher among the women with both MetS and SUD, and the additive risk was significant (*p*-values < 0.05).

**Figure 1:**
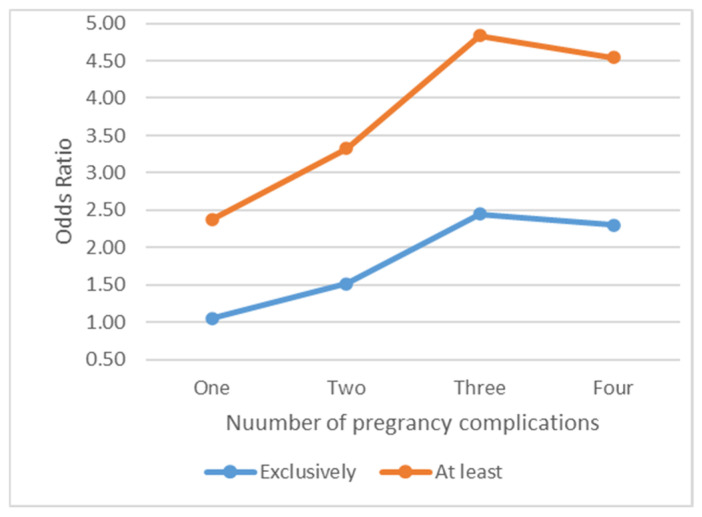
Risks of pregnancy complications among women with both MetS+SUD vs. women with MetS alone.

**Figure 2:**
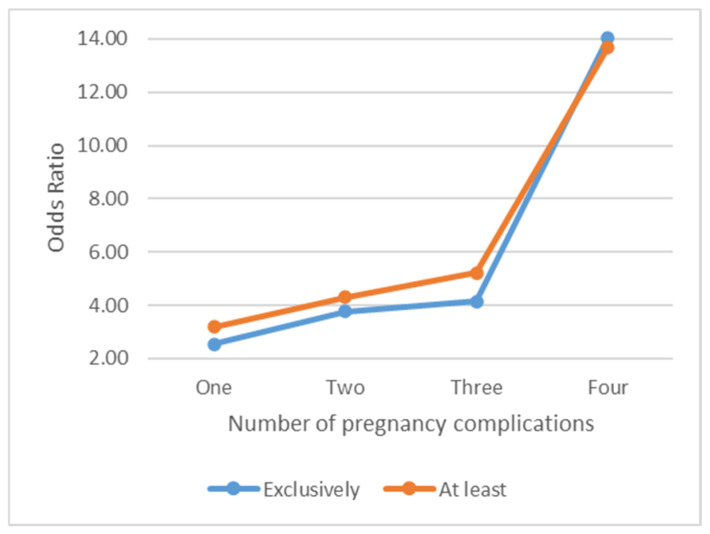
Risks of pregnancy complications among women with both MetS+SUD vs. women with SUD alone.

Similarly, compared to women with SUD alone, women with both MetS and SUD had 218% higher odds of having at least one complication (*p*-value < 0.001), 330% higher odds of developing at least two complications (*p*-value < 0.001), 422% higher odds of developing at least three complications (*p*-value < 0.001), and 1269% higher odds of developing at least four complications (*p*-value < 0.001) (Figure 1 and Figure 2). The consistency of the above results indicates a significantly increased risk of multiple complications among women with both MetS and SUD compared to the women with SUD alone.

Similarly, compared to the women with MetS alone, women with both MetS and SUD had 51% higher odds of developing two pregnancy complications (*p*-value = 0.004), 145% higher odds of developing three pregnancy complications (*p*-value < 0.001), and 130% higher odds of developing four pregnancy complications (*p*-value = 0.002). Compared to women with MetS alone, women with both MetS and SUD had 32% higher odds of having at least one complication (*p*-value = 0.007), 81% higher odds of developing at least two complications (*p*-value < 0.001), 139% higher odds of developing at least three complications (*p*-value < 0.001), and 124% higher odds of developing at least four complications (*p*-value = 0.003). The consistency of the above results indicates a significantly increased risk of multiple complications among women with MetS and SUD both compared to women with MetS alone.

## 4. Discussion

Substance use disorder among pregnant women is a significant public health concern in the United States. The number of pregnant women with opioid use disorder alone at labor and delivery more than quadrupled from 1999 to 2014, according to a recent CDC analysis. Substance use and substance use disorders in pregnancy are common and are linked with multiple adverse obstetric and neo-natal outcomes. Substance use disorder during pregnancy has been linked with serious negative health outcomes for pregnant women and developing babies, including preterm birth, stillbirth, maternal mortality, and neonatal abstinence syndrome (NAS) [27,28]. Similarly, maternal metabolic syndrome in pregnancy, also classified as cardiometabolic risk, is associated with more adverse pregnancy and birth complications [29,30]. More than half of the women who had MetS in early pregnancy developed a pregnancy complication, compared with just over a third of women who did not have MetS [29].

The results of this study consistently show that MetS and SUD can significantly increase the risk of pregnancy complications among pregnant women. Metabolic syndrome amplifies the risk of developing gestational diabetes, preeclampsia, and preterm labor in pregnant women, which in line with other studies mentioned above. Similarly, women with SUD had worse obstetric and birth outcomes, with high prematurity and low birth weight [26], and our results strengthen these findings as well.

Although some findings of obstetric and birth outcomes are similar, in this study, we were able to significantly extend the risk factors not only to MetS, but also to SUD and combinations of both, which occur at a very high rate in Appalachia. Thus, we were able to provide further evidence regarding the pregnancy complications in this highly affected population.

Our results significantly deepen our understanding of pregnancy complications and newborn disorders due to SUD and MetS. We demonstrated that pregnant women who had metabolic syndrome and SUD during pregnancy tended to have a higher risk of pregnancy complications—gestational diabetes, preeclampsia, premature rupture, preterm labor, and newborn disorder—compared to the pregnant women who had MetS alone, SUD alone, or none. This result was consistent. We also found higher odds of developing more complications if the pregnant women had both MetS and SUD. In fact, pregnant women who had MetS and SUD exhibited a very high risk of developing at least four complications compared to those who had neither MetS nor SUD. Our data showed the additive risk of interaction between MetS and SUD, and we observed the significant additive risk of preeclampsia and at least two components. This further necessitates care strategies that could review the adverse outcomes associated with MetS and the most commonly used substances, as well as the need to collaborate with an interprofessional team to counsel, appropriately treat, and support women during their pregnancies for better outcomes.

Considering the increasing cases of MetS and SUD, the results of this study underscore the importance of pregnancy complications due to those factors, particularly in the Appalachian region, which has a higher prevalence of both MetS and SUD. Ideally, all pregnant women should be screened, and those with positive screens should be promptly diagnosed and treated to avoid the morbidity and mortality associated with continued substance use during pregnancy. The provision of counseling on SUD, and other medical treatments to control MetS during pregnancy, may reduce the likelihood of obstetric and newborn problems. However, more evidence is needed in order to determine whether and to what extent such counseling and clinical treatment are effective.

This study has several strengths. First, the use of a large pool of data from the Appalachian region allowed us to investigate the association between MetS, SUD, and obstetric and newborn complications. Another strength of this study is the setting. The population under study was from the Appalachian region, especially from the North Central and Central regions. This region is plagued with a higher prevalence of MetS, obesity, SUD, and numerous other significant health disparities. No such study of this scale, particularly focused on Appalachia, has been conducted in the past. Our study fills that research gap in this highly affected area.

The study also has limitations. The analysis was focused on only the Appalachian region, which could limit the generalizability of our findings to other settings. However, the context of this study fits perfectly for this population with a higher prevalence of obesity and SUD. We also extended the analyses for each risk factor individually—SUD alone and MetS alone—and these findings could be representative of the general population. Another limitation could be due to the small sample size of those with more than three complications; the results for four or at least four complications seem to be inflated. However, the consistency of the results indicates a similar trend. Also, due to the small sample size and data limitations, we were unable to observe other pregnancy complications. Furthermore, in our investigation, we considered overall SUD and MetS and did not investigate different types of SUD or individual MetS components. Further studies are required in order to determine whether individual MetS components act synergistically or independently.

## 5. Conclusions

Both MetS and SUD are associated with higher risks of obstetric and newborn complications compared to pregnant women without these conditions. Among pregnant women with both MetS and SUD, these complications were significantly elevated compared to the pregnant women with MetS alone, SUD alone, or none. The findings from this study highlight the importance of counseling and other forms of clinical treatments to control SUD and MetS during pregnancy.

## Figures and Tables

**Table 1:** Pregnant women, SUD, and complications summary (January 2010–November 2021).

Variables	Average	Sd *
BMI	30.25	7.74
Maternal age at the time of birth	26.40	5.61
**Other Study Variables**	**n**	**%**
Total sample (n = 27,955)		
Obese	9644	34.5
IR	6789	24.29
HTN	3534	12.64
HDL	2439	8.72
TG	1470	5.26
Number of METS criteria met		
0	13,230	47.33
1	8717	31.18
2	3823	13.68
3	1447	5.18
4	518	1.85
5	220	0.79
MetS (≥3 criteria met)	2185	7.82
SUD	6912	24.73
Prescribed opioids (at least once before or at the time of birth)	12,982	46.44
MetS alone (no SUD)	1608	5.75
SUD + MetS	577	2.06
SUD alone (no MetS)	6335	22.66
No MetS or SUD	19,435	69.52
Smoking	11,922	42.65
Alcohol	3496	12.51
Positive HCV antibody test	1483	5.3
HIV-positive	22	0.08
Sepsis	190	0.68
**Outcome Variables**	**n**	**%**
Gestational diabetes	3660	13.09
Preeclampsia	3483	12.46
Premature rupture	2505	8.96
Preterm labor	3165	11.32
Newborn disorder	2278	8.15
Maternal death	4	0.01

* sd—standard deviation.

**Table 2:** Association between MetS, SUD, and pregnancy complications.

Outcome Variables	% of Events				
	MetS Only (n = 1608)	SUD Only (n = 6335)	MetS and SUD (n = 577)	None (n = 19,435)	*p*-Value *
Gestational diabetes	44.65	8.33	41.25	11.2	<0.001
Preeclampsia	23.76	12.3	32.58	10.98	<0.001
Premature rupture	6.97	10.86	8.84	8.51	<0.001
Pre-term labor	10.39	18.12	21.84	8.87	<0.001
Newborn disorder	10.82	14.03	19.24	5.68	<0.001
Maternal death	Not enough data to evaluate				

* *p*-value—based on Chi-square test of association.

**Table 3:** Comparison of odds of pregnancy complications between different groups (MetS alone, SUD alone, both MetS and SUD, and none).

Pregnancy Complications	Comparison Groups															Relative Excess Risk due to Interaction Between MetS and SUD	
	MetS Alone vs. None			SUD Alone vs. None			MetS+SUD vs. None			MetS+SUD Both vs. MetS			MetS+SUD both vs. SUD				
	OR	CI	*p*-Value	OR	CI	*p*-Value	OR	CI	*p*-Value	OR	CI	*p*-Value	OR	CI	*p*-Value	Estimate	*p*-Value (CI)
Gestational Diabetes	6.40	(5.71, 7.09)	<0.0001	0.72	(0.65, 0.79)	<0.0001	5.57	(4.61, 6.52)	<0.0001	0.87	(0.7, 1.04)	0.1575	7.72	(6.27, 9.17)	<0.0001	NA	NA
Preeclampsia	2.53	(2.21, 2.84)	<0.0001	1.14	(1.04, 1.24)	0.0041	3.92	(3.21, 4.62)	<0.0001	1.55	(1.23, 1.87)	<0.0001	3.45	(2.79, 4.1)	<0.0001	1.30	<0.001 (0.51, 2.00)
Premature Rupture	0.80	(0.65, 0.96)	0.0321	1.31	(1.19, 1.43)	<0.0001	1.04	(0.74, 1.35)	0.7807	1.30	(0.85, 1.74)	0.1426	0.80	(0.56, 1.03)	0.1333	−0.07	0.654 (−0.43, 0.28)
Preterm Labor	1.19	(0.99, 1.39)	0.0414	2.27	(2.09, 2.46)	<0.0001	2.87	(2.29, 3.45)	<0.0001	2.41	(1.8, 3.02)	<0.0001	1.26	(1, 1.52)	0.0278	0.41	0.098 (−0.21, 1.02)
Newborn Disorder	2.01	(1.67, 2.35)	<0.0001	2.71	(2.46, 2.96)	<0.0001	3.96	(3.1, 4.81)	<0.0001	1.96	(1.45, 2.47)	<0.0001	1.46	(1.14, 1.78)	0.0007	0.23	0.308 (−0.67, 1.12)
One Complication	2.95	(2.61, 3.3)	<0.0001	1.22	(1.14, 1.3)	<0.0001	3.11	(2.48, 3.75)	<0.0001	1.05	(0.81, 1.3)	0.654	2.55	(2.01, 3.08)	<0.0001	−0.06	0.568 (−0.775, 0.651)
Two Complications	4.47	(3.75, 5.19)	<0.0001	1.80	(1.61, 1.99)	<0.0001	6.77	(5.1, 8.44)	<0.0001	1.51	(1.09, 1.94)	0.004	3.76	(2.8, 4.71)	<0.0001	1.50	0.048 (−0.26, 3.25)
Three Complications	4.71	(3.54, 5.88)	<0.0001	2.79	(2.36, 3.21)	<0.0001	11.55	(8.01, 15.09)	<0.0001	2.45	(1.55, 3.36)	<0.0001	4.15	(2.85, 5.44)	<0.0001	5.05	0.002 (1.50, 8.61)
Four Complications	10.86	(6.54, 15.17)	<0.0001	1.78	(1.09, 2.47)	0.00	24.98	(13.26, 36.69)	<0.0001	2.30	(1.09, 3.51)	0.002	14.04	(6.75, 21.33)	<0.0001	13.34	0.010 (2.09, 24.59)
Atleast One Complications	3.43	(3.07, 3.79)	<0.0001	1.43	(1.35, 1.51)	<0.0001	4.54	(3.72, 5.36)	<0.0001	1.32	(1.05, 1.6)	0.007	3.18	(2.59, 3.77)	<0.0001	0.68	0.064 (−0.20, 1.57)
Attleast Two Complications	4.83	(4.15, 5.5)	<0.0001	2.03	(1.86, 2.21)	<0.0001	8.74	(6.9, 10.57)	<0.0001	1.81	(1.37, 2.25)	<0.0001	4.30	(3.37, 5.22)	<0.0001	2.87	0.001 (0.98, 4.78)
Atleast Three Complications	5.72	(4.49, 6.94)	<0.0001	2.61	(2.24, 2.99)	<0.0001	13.65	(9.93, 17.36)	<0.0001	2.39	(1.62, 3.15)	<0.0001	5.22	(3.77, 6.68)	<0.0001	6.31	<0.001 (2.60, 10.03)
Atleast Four Complications	10.73	(6.53, 14.93)	<0.0001	1.76	(1.09, 2.43)	0.00	24.04	(12.81, 35.27)	<0.0001	2.24	(1.07, 3.42)	0.003	13.69	(6.62, 20.76)	<0.0001	12.56	0.011 (1.74, 23.37)

OR—odds ratio, CI—95% confidence interval, NA—not applicable.

## Data Availability

We used electronic health data from a hospital system with identifiers that cannot be shared in their current form due to privacy or ethical restrictions. But we are glad to share the aggregated summary and results with other researchers. Also, if somebody requests the deidentified data with limited fields used in this study, we will be able to share a part of this dataset, as we are using the same dataset for other research works and cannot share the entire dataset. Please contact the corresponding author if you would like to request a report or partial data.
